# Dynamic Associations of Change in Physical Activity and Change in Cognitive Function: Coordinated Analyses of Four Longitudinal Studies

**DOI:** 10.1155/2012/493598

**Published:** 2012-09-16

**Authors:** Magnus Lindwall, Cynthia R. Cimino, Laura E. Gibbons, Meghan B. Mitchell, Andreana Benitez, Cassandra L. Brown, Robert F. Kennison, Steven D. Shirk, Alireza Atri, Annie Robitaille, Stuart W. S. MacDonald, Elizabeth M. Zelinski, Sherry L. Willis, K. Warner Schaie, Boo Johansson, Marcus Praetorius, Roger A. Dixon, Dan M. Mungas, Scott M. Hofer, Andrea M. Piccinin

**Affiliations:** ^1^Department of Food and Nutrition, and Sport Science and Department of Psychology, University of Gothenburg, P.O. Box 300, 405 30 Gothenburg, Sweden; ^2^Department of Psychology and Neurology, University of South Florida, 4202 East Fowler Avenue, Tampa, FL 33620, USA; ^3^General Internal Medicine, Harborview Medical Center, University of Washington, 325 Ninth Avenue, P.O. Box 359780, Seattle, WA 98104, USA; ^4^Massachusetts General Hospital, Harvard Medical School, ENRM Bedford VA Hospital, 200 Springs Road, Bedford, MA 01730, USA; ^5^Center for Biomedical Imaging, Medical University of South Carolina, 68 President Sreet, MSC 120, Charleston, SC 29425, USA; ^6^Department of Psychology, University of Victoria, P.O. Box 3050 STN CSC, Victoria, BC, Canada V8W 3P5; ^7^Department of Psychology, California State University, Los Angeles, 5151 State University Drive, Los Angeles, CA 90032, USA; ^8^Andrus Gerontology Center, Leonard Davis School of Gerontology, University of Southern California, Los Angeles, CA 90089, USA; ^9^Department of Psychiatry and Behavioral Sciences, University of Washington, 180 Nickerson, Suite 206, Seattle, WA 98109, USA; ^10^Department of Psychology, University of Gothenburg, P.O. Box 100, 405 30 Gothenburg, Sweden; ^11^Department of Psychology, University of Alberta, Edmonton, AB, Canada T6G 2E9; ^12^Davis Lawrence J. Ellison Ambulatory Care Center, University of California, 4860 Y Street, Ste 0100, Sacramento, CA 95817, USA

## Abstract

The present study used a coordinated analyses approach to examine the association of physical activity and cognitive change in four longitudinal studies. A series of multilevel growth models with physical activity included both as a fixed (between-person) and time-varying (within-person) predictor of four domains of cognitive function (reasoning, memory, fluency, and semantic knowledge) was used. Baseline physical activity predicted fluency, reasoning and memory in two studies. However, there was a consistent pattern of positive relationships between time-specific changes in physical activity and time-specific changes in cognition, controlling for expected linear trajectories over time, across all four studies. This pattern was most evident for the domains of reasoning and fluency.

## 1. Introduction

Previous research has clearly demonstrated that cognitive change in old age does not occur in a homogenous manner for all individuals [1–3]. A number of predictors of cognitive change in old age have been identified, such as education, hypertension, objective indices of health and cardiovascular disease, and apolipoprotein E [4]. Regular engagement in different types of activities may also influence cognitive change. More specifically, according to the “use it or lose it” hypothesis [5], regular engagement in different activities may buffer age-related decline in cognitive functioning. A number of studies have found that general lifestyle activity engagement (often operationalized as the combination of intellectual, social, and physical activities) is associated with cognitive change [6–8] and that decline in activity in older age is associated with decline in cognitive functioning.

In addition to general activity, other studies have specifically targeted the association of physical activity with cognitive change. Indeed, a growing body of the literature highlights the potential benefits of physical activity on the structure and function of the brain [9, 10]. The first line of evidence for the relationship between physical activity and cognition comes from a number of cross-sectional studies demonstrating that physically active older adults have higher cognitive performance and functioning compared with less active older adults [11, 12].

However, the evidence derived from these cross-sectional studies is limited, as it is not. possible to draw conclusions in terms of more complex associations of change. Stronger evidence may be found in longitudinal studies. Longitudinal studies may be viewed as the second line of evidence, offering valuable information on the relationship between physical activity and cognition across time. Several prospective, longitudinal studies provide evidence for the association of physical activity with cognitive functioning [13–20]. These studies have generally shown that higher physical activity at baseline is associated with less decline in cognitive functioning over time, offering support for the notion that regular physical activity may buffer against future cognitive decline. However, results from these longitudinal studies are inconclusive [4] and several critical questions remain [21]. 

The longitudinal studies described above may test two different classes of hypotheses regarding the relationship of lifestyle variables, such as physical activity and cognitive change [6]. The first type of hypothesis stipulates that the level of physical activity is related to subsequent cognitive change. The majority of the abovementioned studies have targeted this first class of hypothesis, examining a stable change hypothesis by looking at how physical activity at baseline predicts change in cognitive functioning. The second class of hypothesis instead examines the relationship between concomitant change in activity and change in cognition. In contrast to the baseline effect of activity, this hypothesis deals with the concept of intraindividual change and associations among intraindividual rates of change, providing a more dynamic perspective. For example, positive changes (increases) in physical activity across time may be hypothesized to contribute to a less negative change (less decline) in cognitive functioning, whereas a negative change in activity (decreased activity) would be expected to be related to faster cognitive decline with age. 

Mackinnon and colleagues [7] used a latent growth curve modeling approach to examine how change in overall activity (defined as a composite of physical activity, rest, interest and hobby related, and planned activities), rather than physical activity, was related to change in health and cognitive performance. They found substantial correlations between rates of change in activity and cognitive and health measures, and it was concluded that decline in mental and physical activity in older age is paralleled by decline in cognitive functioning and health. However, decline in cognitive functioning was still evident for participants who were stable in their level of activity across time, suggesting that maintenance of activity may not be enough to protect from cognitive decline.

Unfortunately, few previous studies have actually targeted the hypothesis of whether there are associations among rates of individual change in physical activity and cognitive functioning in long-term observational studies of aging individuals. Van Gelder and colleagues [17] found that men who decreased their physical activity duration or intensity also demonstrated a stronger decline in overall cognition (measured by the Mini Mental State Examination) compared with men who maintained their activity duration and intensity. However, several limitations should be noted in this study. First, only change in one global measure of cognitive functioning was used, rather than several different measures that may capture more diverse and complex relationships of cognitive ability with activity change. Moreover, in the study, change in physical activity was categorized in terms of quality (change/no change) rather than quantity (how much change). Finally, the analyses were based on between-group comparisons and therefore did not target relationships of within-person changes in physical activity and cognition. 

Bielak and colleagues [22], however, used random effects models to examine how level and change in physical activity were related to level (within-person mean) and inconsistency (within person standard deviation across trials) of cognitive speed at baseline and change in level and inconsistency. Although physical activity at baseline was related to mean cognitive speed in some tasks, there were no associations between change in physical activity and change in cognitive speed. Moreover, a recent study [23] using bivariate dual-change-score models to analyze data from the Victoria Longitudinal Study found that changes in physical activity influenced changes in verbal speed and episodic memory. However, they also found that changes in cognition influenced changes in activity, thus supporting a dual-coupling model or a reciprocal relationship between physical activity change and change in cognition. 

Although previous longitudinal studies have resulted in increased understanding of how physical activity at one point (baseline) may predict future cognitive performance, or change in cognitive performance, they have generally not helped us understand the more complex and dynamic characteristics of the longitudinal relationship between physical activity and cognition. Relevant questions remain unanswered. For example, is there an association between within-person change in physical activity and within-person changes in cognitive functioning when taking into account the change in cognition due to time? Or, put differently, do persons demonstrate lower cognitive scores (relative to their *within-person *trajectory over time) on occasions when they also report less physical activity? Relative to a cross-sectional analysis that compares individuals to other similar aged individuals, the answers to these questions afford relevant insight into the more complex and dynamic patterns of associations between changes in physical activity and cognitive functioning within individuals across time. Another question that has not been properly addressed by previous longitudinal studies is if physical activity, or change in activity, has similar effects across different cognitive domains and/or tests. From previous experimental work using randomized controlled trial designs, there is support for the notion that physical activity training has the strongest effect on executive control processes and working memory, supporting the “selective-improvement hypothesis” [24]. However, as the majority of previous longitudinal studies of the relations between physical activity and cognition have included a single measure of cognition (often MMSE) rather than different tests and domains, the theoretically, as well as practically, important question of whether changes in physical activity may relate more strongly to changes in some cognitive domains relative to others remains unresolved.

An essential step for the sound cumulative development of this body of knowledge is the reproduction and extension of research findings across independent longitudinal studies that focus on observed within-person change [25]. Although most previous longitudinal studies have found that physical activity is protective against age-related cognitive decline, the findings are disparate and far from clear [21]. Moreover, previous studies have typically used data from one population (e.g., adults ranged from 55–94 in age) and one design (e.g., 3 waves of measurements over a 6-year period), leaving the generalization of the results highly contingent on sample specific-characteristics. Differences between studies in terms of sample, design, measures, and analytical approach make it difficult to compare results across studies and to derive more general conclusions of the meaning of these results. Therefore, there is a clear need for more coordinated integrative data analyses that use data from different samples with different measures, but examine these data with the same research question and the same analytic approach [25]. Using a coordinated analysis approach for cross-study comparisons and synthesis of independent results has the potential to bring new relevant information to the field of cognitive change and physical activity [26]. 

The purpose of the present study is to investigate the longitudinal associations of physical activity with four domains of cognition (i.e., reasoning (executive function), episodic memory, fluency, and semantic knowledge) in older adults using a coordinated approach with data from four independent longitudinal studies: Long Beach Longitudinal Study (LBLS), the Seattle Longitudinal Study (SLS), the Victoria Longitudinal Study (VLS), and the Origins of Variance in the Oldest-Old: Octogenarian Twins Study (OCTO-Twin). More specifically, the following research questions were examined:

- Is physical activity at baseline associated with cognition at baseline?
- Is baseline physical activity associated with the rate of change in cognition?
- Are occasion-specific changes in physical activity associated with occasion-specific changes in cognition, controlling for change in cognition due to time alone?

## 2. Methods

### 2.1. Design and General Analytical Framework

This research, initiated as a partnership between the Advanced Psychometric Methods Workshop series (Mungas et al., NIA conference grant) and the Integrative Analysis of Longitudinal Studies on Aging (IALSA) network [25, 26], brought workshop participants together with researchers from four IALSA member studies. These studies were specifically selected based on their collection of cognitive, physical, and social activity data along with a range of cognitive functioning measures over multiple occasions held in common across the four studies. While the activity and cognitive functioning variables are not always identical, the subsets of variables in each study were chosen based on the rationale that they tapped similar domains at the construct level (e.g., fluid reasoning (Gf), crystallized knowledge (Gc), short-term memory (Gsm,) and long-term storage and retrieval (Glr; category fluency) [27]. In some cases the measures are the same, but more often they differ, providing opportunities for both strict and conceptual replication. 

### 2.2. OCTO-Twin (Origins of Variance of the Oldest-Old) Sample (Sweden)

 The OCTO-Twin study is comprised of the oldest-cohort of the Swedish Twin Registry aged 80 and older at the time of first examination. Beginning in 1991–93, the longitudinal design included a maximum of five measurement occasions at 2-year intervals. Individuals with a dementia diagnosis at baseline (*n* = 98) were excluded from the initial sample of 702 participants. The remaining 604 individuals were included in analyses. Approximately 20% of the sample was lost to follow up at each wave (10% per year), but most of this attrition was due to death. Descriptive statistics are provided in Table 1.

### 2.3. OCTO-Twin Materials and Procedures

#### 2.3.1. OCTO-Twin Cognitive Ability Measures

*Reasoning* was assessed using Block Design [28]. In this task, participants were presented with red and white blocks and instructed to reproduce the design shown on a card using these blocks within a predetermined time limit. *Fluency* was not assessed in the OCTO-Twin Study. *Memory *was assessed using immediate recall of the Prose Recall test, in which participants were presented with a brief, 100-word story that had a humorous element [29]. Amount of information recalled was coded in a manner similar to the scoring of story units in the Wechsler Memory Scale Logical Memory test [30]. *Semantic knowledge *was assessed using the Swedish version of the Information Task, in which participants provided responses to factual knowledge questions [31]. Raw scores were transformed into T-scores with a mean of 50 and standard deviation of 10 to facilitate comparisons across measures. 

#### 2.3.2. OCTO-Twin Physical Activity Measure

Respondents were asked, at each of the five waves, the following: *“Are you presently doing or have you previously done anything special to train your body or keep your body fit?”* The possible responses were “no” (0), “yes, to some extent” (1), or “yes, to a great extent” (2). Hence, a scale from 0 to 2 was used. The participants gave one reply for their present physical activity status and one in regards to their previous status. Only the answer for the present status was used in the analyses. Physical activity change scores were computed by subtracting baseline activity from each follow-up activity measure. 

### 2.4. Long Beach Longitudinal Study Sample (California, USA)

The LBLS was initiated in 1978 when participants were recruited from the Family Health Plan Health Maintenance Organization (HMO), mainly including residents of Long Beach and Orange County. This first panel included 583 individuals aged 28–36 or 55–87. The ethnic composition of the older group (98% Caucasian) was similar to the 65+ population for the area based on the 1970 census. Panel 2, initiated in 1992, included 633 individuals from the same HMO (64 were excluded due to frank dementia or serious sensory or neurological problems).

In order to include the same measures as those in the Seattle Longitudinal Study, LBLS Panel 1 (*n* = 106) and Panel 2 (*n* = 631) data from 1994 to 2003, excluding participants younger than age 55 in 1994 (*n* = 541), were used in the current analysis. During this period, data were collected at 3-year intervals. Attrition was approximately 50% over each interval or 17% per year. Dementia incidence is not known. Descriptive information for the sample is presented in Table 2.

### 2.5. LBLS Materials and Procedures

#### 2.5.1. LBLS Cognitive Ability Measures

*Reasoning* was based on a composite score of the Letter and Number Series tests from the Schaie-Thurstone Adult Mental Abilities Test (STAMAT; [32]. In this task, participants viewed a series of letters (e.g., a b c c b a d e f f) and were asked to identify the next letter in the series from alternate choices by extracting the rule that governed the series. Responses were made by choosing the correct alternative from an array of possible alternatives. Participants were given six minutes to complete as many of the 30 items as possible. *Fluency *was assessed using Word Fluency, in which participants wrote down as many words as possible that begin with the letter “s” during a five-minute period according to predetermined rules. These rules included no proper names and no addition of endings to words that the participant had already provided (e.g., “sit,” “sitting,” and “sits”). *Memory* was measured using immediate written recall of a 20-item noun list that participants had studied for 3.5 minutes. *Semantic knowledge* was assessed using the STAMAT Recognition Vocabulary test. Participants were provided with 50 target words and asked to select the synonym from four choice alternatives. Performance was based on total correct responses provided in a five-minute period. 

#### 2.5.2. LBLS Physical Activity Measure

The physical activity measure was based on selected questions from the Life Complexity Scale. A composite score was created by summing the number of physical activities (e.g., walking, outdoor hobbies, etc.) that included one or more hours of these activities per week. The range of possible scores was from 0 to 4. Activity change variables were computed by subtracting the activity measure in 1994 from activity in 1994, 1997, 2000, and 2003. This resulted in difference scores that were referent to the baseline testing in 1994.

### 2.6. Seattle Longitudinal Study Sample (Washington, USA)

The SLS is a long-running longitudinal study initiated by K. Warner Schaie, who first recruited members of a local Health Maintenance Organization in 1956. Current analyses used up to four waves of SLS data from 1984–2005, which include an expanded set of measures that also overlapped with the Long Beach Study. Only participants 55 years and older at baseline were included in the analysis. Baseline was defined as each participant's first study visit, and time was measured in all analyses as years in study (coded as 0, 7, 14, and 21). Attrition during these 7-year intervals was approximately 50% or 7% per year. Dementia prevalence and incidence are not known. See Table 3 for SLS participant characteristics over the four waves of data analyzed here.

### 2.7. SLS Materials and Procedures

#### 2.7.1. SLS Cognitive Measures

*Reasoning* was assessed with the Word Series test from the Schaie-Thurstone Adult Mental Abilities Test (STAMAT; [32]). In this task, participants were provided with a printed word series that was ordered according to an inherent rule. The participant's task was to select, from multiple-choice options, the next word in the series consistent with that rule. Total score was based on number of correct responses to the 30 trials completed in 6 minutes. As in LBLS, *Fluency *was indexed by performance on the Word Fluency Test from the Primary Mental Abilities test [33] and *Memory* by the verbal list-learning task. *Semantic knowledge *was assessed with the test of Advanced Vocabulary from the Educational Testing Service (ETS), in which participants identified synonyms for printed words from five choices [34]. The total score was derived from the number of correct responses provided within 4 minutes to the 36-itemtest. 

#### 2.7.2. SLS Physical Activity Measure

The methodology described in the LBLS method portion of this paper was used in order to generate roughly equivalent indices of physical activity. Following this methodology, a composite physical activity measure was created by summing dichotomized test responses from a modified version of the Life Complexity Scale [3], resulting in a four-item physical activity composite (playing sports, walking, fitness, and outdoor hobbies). *Activity change* was computed by subtracting baseline activity from each follow-up activity measure. 

### 2.8. Victoria Longitudinal Study Sample (British Columbia, Canada)

The Victoria Longitudinal Study began in 1986–87 with a sample of 484 community residing volunteers. Using a longitudinal sequential design, second and third independent samples began in 1992-93 (*n* = 530) and 2001-2002 (*n* = 550) [35]. Each sample is tested at three-year intervals. To date, Sample 1 has been tested on seven occasions (over 18 years), Sample 2 on five (over 12 years), and Sample 3 on two occasions (over 6 years). Participants in all three samples were recruited between the ages of 55 and 85 years. 

Data from seven waves of Sample 1 and five waves of Sample 2 were included in the current investigation. Characteristics of the subsample analyzed here are provided in Table 4. Approximately 20% of the sample was lost to follow up at each wave, or 10% per year. Dementia prevalence and incidence are not known.

### 2.9. VLS Materials and Procedures

#### 2.9.1. VLS Cognitive Ability Measures

*Reasoning* was indexed by Letter Series [33]. In this task, participants were presented with a series of letters and asked to identify the next letter in the sequence based on the rule that governed the sequence. *Fluency* was measured by performance on a Similarities task [34], in which participants were presented with target words and asked to write as many words as possible with the same or nearly the same meaning during a 6-minute period. *Memory *was indexed based on free recall of a 30-item noun list comprised of five semantic categories. Participants were given two minutes to study the words and then five minutes to recall them [35]. *Semantic knowledge* was assessed using a 54-item recognition vocabulary test adapted from the ETS Kit of Factor Referenced Tests [34]. 

#### 2.9.2. VLS Physical Activity Measure

The physical activity measure was derived from a subset of four items from the VLS- Activity Lifestyle Questionnaire (VLS-ALQ; [6]). These items indexed the physical activities of gardening, jogging, sailing, and tennis. For each item, participants indicated the frequency of engagement in that activity over the past two years on a scale from 0 to 9 (i.e., *never, less than once a year, about once a year, 2 or 3 times a year, about once a month, 2 or 3 times a month, about once a week, 2 or 3 times a week, and daily)*.

### 2.10. Analytic Approach

In order to examine the effects of physical activity on cognition, a series of multilevel models was fit with time varying covariates [36] using multilevel mixed-effects regression in Stata (StataCorp, 2011), the restricted maximum likelihood estimator (REML), and an unstructured covariance matrix. Separate models were fit for each of the four cognitive measures (reasoning, fluency (except OCTO-Twin), memory, and semantic knowledge) and for each of the four studies. In order to improve ease of interpretation of our results, age, education, and activity measures were mean centered to the baseline mean of each measure in the sample so that the intercept and linear slope terms could be interpreted as the expected value for an individual at the mean age, education, and respective activity level at baseline. The reference category for sex was male. OCTO-Twin participants were modeled as nested within their twin pair and in VLS we controlled for enrolment cohort.

Our goal was to build a common model for comparisons across all outcomes for the four longitudinal studies. This common model was not necessarily the optimal model for each of the 16 cognition-physical activity combinations. An initial 19-term model included all ten two-way interactions that included activity, change in activity or time, and three 3-way interactions of time and activity with age, sex, or education. However, several terms were not significant for most of the studies and outcomes and so were trimmed to facilitate model interpretation. First, the 3-way interactions were eliminated, then the interactions with change in activity. Last, the baseline activity by sex interaction was dropped. This resulted in a final model that included 12 terms summarized in Table 5 for separate cognitive constructs of reasoning, memory, semantic knowledge, and fluency. Our significance criterion of *P* < 0.05 shaped the “familywise” alpha rate within each study, however our focus was on the repetition of results across studies, which we used to minimize the potential impact of chance associations. We did not implement formal meta-analytic techniques as they require identical measures and a larger number of studies.

## 3. Results

### 3.1. Baseline Covariates and Longitudinal Relationships

Between-person age differences are seen at the first occasion of measurement for all memory, reasoning, and fluency tests, with older adults performing less well. Semantic knowledge results are less consistent, with older adults performing less well in LBLS and OCTO-Twin, but better in VLS. At baseline, individuals with more years of education had higher cognitive performance. LBLS and SLS women scored higher than men of the same age on all measures, except for semantic knowledge. OCTO-Twin and VLS women had higher memory scores than did the men. OCTO-Twin women had lower scores on semantic knowledge.

For the reference individuals (men with sample average baseline age and years of education), within-person declines were seen over time in all cognitive abilities and all samples except SLS (the youngest), where the relationship, as in LBLS and VLS, depended on age. Older individuals declined faster on all VLS, SLS, and LBLS measures except LBLS immediate memory. No clear pattern was identified in regards to differential decline related to sex or education. 

### 3.2. Physical Activity

Higher physical activity at baseline was associated with higher scores on reasoning and memory in OCTO-Twin and VLS and fluency in SLS. The association between physical activity score at baseline and cognitive score did not differ by age. However, there was some indication that the relation between physical activity and cognition differed by education. For semantic knowledge in LBLS and SLS the association with physical activity at baseline was stronger for people with less education. In terms of associations with cognitive decline, higher physical activity score at baseline was related only to less decline on fluency in VLS and SLS. 

However, we found a consistent pattern of positive relationships between time-specific changes in physical activity and time-specific changes in cognition beyond those expected by the estimated linear trajectories in the four studies. This pattern was most evident for the domains of reasoning and fluency. More specifically, after controlling for the trend in cognitive functioning over time, time-specific changes in physical activity change were related to cognitive fluctuations in the following cognitive domains: (a) reasoning in all four studies; (b) fluency in two (VLS and SLS) of three studies; (c) memory in two studies (OCTO-Twin and VLS); (d) semantic knowledge in one study (OCTO-Twin). 

## 4. Discussion

Using data from four longitudinal studies of aging, the present study examined the relationship between physical activity and cognitive functioning at three different levels: (a) cross-sectionally; (b) longitudinally using physical activity as predictor of cognitive change; (c) longitudinally using change in physical activity as a time-varying covariate to predict change in cognition, adjusting for the normative development (effect of time) in cognition. On the cross-sectional level, higher physical activity at baseline was associated primarily with higher scores on reasoning and fluency, generally supporting previous studies demonstrating that physically active older adults have higher cognitive performance and functioning compared with less active older adults [11, 12]. Although relevant, these well-known and well-documented findings contribute little to a deeper understanding of the likely very complex relationship between activity and cognition.

The second level of analysis addressed more theoretically relevant and interesting longitudinal associations and the question of whether physical activity at baseline is associated with cognitive change. From a broader perspective, this research question is also linked to the “use it or lose it” hypothesis [5, 21], generally proposing that physical activity may buffer against future cognitive decline. A number of prospective studies have found support for this notion [13–20], offering preliminary support for the longitudinally beneficial and buffering effect of physical activity on cognitive decline. A general limitation in many of these previous prospective studies, however, is that they have examined the association between physical activity and a broad global measure of cognition (typically MMSE), thereby proving an incomplete picture of the potentially diverse longitudinal associations between different cognitive domains and physical activity. As a consequence, these previous studies generally have not answered the theoretically and practically relevant question “what cognitive domains most benefit from physical activity?” [21]. 

In the present study four different domains were examined, representing a broader spectrum of cognitive abilities, ranging from the more crystallized knowledge-based domain of semantic knowledge to more fluid or process-based factors of reasoning, fluency, and memory. Higher baseline physical activity was associated with less fluency decline in two of three studies. Thus, the preliminary answer to the question what cognitive domain benefits most from physical activity, based on the results of the present study, is fluency, which is one of the more process-based/fluid domains. However, it should be mentioned that for most cognitive domains across the four studies, we did not find support for the protective effect of baseline levels of physical activity on cognitive decline.

Aside from the more stationary change relationships typically investigated in previous studies (how level of physical activity at baseline relates to change in cognition), we also targeted more dynamic associations between changes in physical activity and changes in cognitive functioning by using change in physical activity from baseline as a time-varying covariate in longitudinal multilevel models. The time-varying covariate model used in this study examined occasion-specific intraindividual relations between physical activity and cognition, after controlling for individual rates of change over time. Such time-specific associations between fluctuations in activity and cognition have rarely been examined and may be highly relevant to understanding how to prescribe exercise and how to design and implement interventions including physical activity to optimize effects on cognition [37]. As associations of change and time-specific fluctuations are of key importance in the analyses and interpretation of intervention studies, the results of studies like the present one may offer new relevant information both from a scientific as well as from an applied perspective. 

 The results from these analyses were surprisingly consistent across studies and domains. Variation in physical activity was associated with variation in reasoning in all four studies and in fluency in two of three studies. Hence, although evidence for the association of between-person differences in baseline physical activity with subsequent cognitive change was generally weak across domains, aside from fluency, support for the notion that change in physical activity covaries with fluctuations in cognition is much more robust across studies. These results are inline with previous work [16], indicating that physical activity may specifically moderate the decline in cognitive domains that is typically associated with aging. Moreover, from a broader perspective, the stronger associations across time of physical activity with more fluid cognition may be linked to the hypothesis that exercise and aerobic fitness training results in selective improvement in executive control processes and working memory [24]. Although the cognitive tests used in the present study to measure reasoning and fluency were derived from a psychometric tradition of psychological testing and do not map well onto more recent conceptualizations of executive processing, they do share some of these features, being a more fluid measure of cognition. 

An obvious limitation of the current study is the observational nature of the longitudinal designs, making inferences in terms of cause and effect irresolvable. The notion that decline in cognition leads to decline in physical activity is equal in validity to the interpretation that a decline in physical activity leads to a decline in cognition, that the relationship is reciprocal, or that both are a result of some third variable. Recent studies provide evidence for not only the reciprocal relationship across time between physical activity and mental health in older adults [38], but also for the reciprocal relationship between physical activity and cognition [23]. 

 Another limitation is the problem associated with using different tests in different studies to tap the same cognitive domains. As noted earlier, the studies were selected because they shared similar measures of activity and cognition. Moreover, single cognitive tests are always imperfect markers of a cognitive domain. Therefore, a general feature of the integrated analytical approach in the present study, where data and tests from four different studies are used to answer the same research question, is the risk of heterogeneity in terms of how well the different tests indicate the higher order construct they should measure. As a result, when patterns of results are not consistent across studies, additional questions, testable in future research, are raised with respect to the source of these differences. It is, for example, interesting to note that for OCTO-Twin, in which physical activity was operationalized as the extent to which persons saw themselves as purposefully “keeping their body fit,” baseline activity was associated with cognitive functions more consistently than were the physical activity measures in the other studies. The apparent importance of physical activity, however, may also be due to the more advanced age of this sample. In situations where the studies with identical measures agree with each other, but not the remaining studies, for example, where neither SLS nor LBLS shows associations between memory performance and either baseline or change in activity, but OCTO-Twin and VLS do, we may draw conclusions that something about the measurement is important. In contrast, LBLS and SLS fluency results do not agree, suggesting instead that some detail relevant to the sampling, retest interval, or other study characteristic may be relevant.

On the other hand, when patterns of results do show consistency across studies that have used different measures to tap the same underlying construct, such as the association of change in activity with change in reasoning and fluency, these differences become a major strength, as the reliability of the conclusions drawn is considerably strengthened compared with traditional analysis of a single dataset. 

Moreover, in contrast with the more specific measurement of cognition, the physical activity variables used in the current analyses were broad and self-reported and did not differentiate aerobic from strength or resistance training. Combining objective measures of physical activity with more specific multi-item, self-report instruments would likely provide future studies with a more robust base for the analysis of the association of change in activity with change in cognition.

The dynamic associations between physical activity and cognitive functioning underscore the broader question of associations between biological and cognitive aging [39]. Also, the effect of intraindividual change in physical activity on cognitive functioning (adjusting for the trend in cognition) raises the question of what drives, or causes, these relationships across time? These dynamic associations with the more fluid cognitive domains may be mediated, or explained, by a number of factors [40], such as physical resources (sleep, energy/fatigue, appetite, pain, or drug/medication use), disease states (hypertension, diabetes, and CVD), and mental resources (chronic stress, depression, and self-efficacy). More specifically, a number of physiological mediators, such as aerobic fitness, hormones, lipid profiles, cerebral blood flow, blood pressure, neurotransmitters, and neurotrophins have all been identified as potential mediators in physical activity-cognition relationships [41]. Although intuitively appealing and quite frequently investigated, the hypothesis of physical activity leading to improved cognition via increased aerobic fitness (the cardiovascular fitness hypothesis) is not, however, supported by meta-analyses [24, 42]. Thus, although single mediation models are theoretically attractive, and may fit data to some extent, the more complete pathways explaining why physical activity and cognition seem to change together more likely include multiple mediators and complex micromediational chains [41], that also may vary in strength and validity across individuals and groups. Nevertheless, increasing knowledge about what precise mechanisms are active ingredients in the effects of physical activity on cognition constitutes a vital step towards the development of appropriate physical activity intervention designs to test these specific models of mediation and the effects of physical activity and exercise on cognition in experimentally controlled trials. 

The major strength of the present study is the ability to elucidate consistent patterns of complex associations across time through coordinated analyses of data from four longitudinal studies. Contrary to previous research based on analyses of single samples, which are limited by the specific characteristics of the sample and data, we instead used a coordinated and integrated analytical approach and framework [25, 26] to examine the same research question in data from four longitudinal studies, thereby making the conclusions less vulnerable to study specific characteristics. As such, the present study is unique (in particular considering the choice of analytical approach) and may pave the way for similar collaborative projects where the same research question and analytical approaches are used to answer relevant questions simultaneously across different studies linked to the association of lifestyle, physical activity, and cognitive functioning.

 The four studies included afford considerable heterogeneity in terms of age (ranging from mean age of 67 in SLS to 83 in OCTO-Twin), number of available waves of measurement (four in SLS and LBLS to seven in VLS), years of followup (8 years in OCTO-Twin to 21 years in SLS), years between measurements (every 2 years in OCTO-Twin to every 7 year in SLS), and cultural background (Scandinavia to North-America). Yet, as discussed above, a surprisingly clear pattern emerged across studies in the relation of change in activity to fluctuations in cognition. Thus, in terms of capacity to identify patterns of associations from a larger and broader perspective and to be able to generalize results and conclusions, the present study brings reproduced evidence to the field as well as to practitioners working with health related behavior, lifestyle, and cognition in elderly. Based on the results in the present study, the main message is that change in activity, and not only previous or current level of activity, seems to matter and may play a significant role in the pursuit of maintaining benign nondecreasing trajectories of cognition along the path of cognitive aging. 

## Figures and Tables

**Table 1:** OCTO-Twin participant characteristics.

Measure	Year of testing				
	Baseline (*N* = 574)	Year 2 (*N* = 471)	Year 4 (*N* = 363)	Year 6 (*N* = 275)	Year 8 (*N* = 201)
	M (SD)	M (SD)	M (SD)	M (SD)	M (SD)
Retention from previous testing (%)		82.0	77.1	75.8	73.1
Age	83.3 (3.0)	85.2 (2.8)	86.9 (2.5)	88.8 (2.5)	90.7 (2.4)
Education	7.3 (2.4)	7.3 (2.3)	7.3 (2.3)	7.2 (2.1)	7.2 (2.2)
Sex, female (*n* (%))	371 (65)	346 (65)	236 (65)	196 (71)	148 (74)
Reasoning	11.5 (7.1)	11.4 (7.2)	11.3 (7.1)	10.8 (7.2)	10.0 (7.3)
Memory	9.6 (4.0)	9.4 (4.3)	9.2 (4.4)	9.2 (4.7)	8.9 (4.5)
Semantic knowledge	28.1 (11.2)	28.6 (11.2)	27.5 (12.4)	26.7 (13.0)	26.1 (11.4)
Physical activity	0.7 (0.7)	0.9 (0.7)	0.8 (0.7)	0.7 (0.7)	0.6 (0.6)
Physical activity change^a^		0.1 (0.7)	−0.0 (0.8)	−0.1 (0.8)	−0.3 (0.9)

M: mean, SD: standard deviation. The scoring ranges for each measure with a defined upper limit are as follows: reasoning: 0–42; memory: 0–16; semantic knowledge: 0–44; physical activity: 0–2. ^a^Physical activity change represents change from baseline.

**Table 2:** LBLS participant characteristics.

Measure	Year of testing			
	Baseline (*N* = 541)	Year 3 (*N* = 275)	Year 6 (*N* = 140)	Year 9 (*N* = 94)
	M (SD)	M (SD)	M (SD)	M (SD)
Retention from previous testing (%)		50.8	50.9	67.1
Age	73.6 (9.0)	75.1 (8.5)	75.1 (8.0)	75.9 (7.1)
Education	13.8 (3.0)	13.9 (2.9)	14.3 (2.7)	14.2 (2.7)
Sex, female (*n* (%))	258 (51)	137 (50)	71 (51)	45 (48)
Reasoning	22.5 (11.7)	24.1 (11.4)	25.4 (11.5)	25.3 (10.7)
Fluency	32.7 (11.4)	33.7 (11.2)	33.6 (12.9)	34.5 (11.8)
Memory	11.4 (4.0)	11.7 (4.3)	11.5 (4.5)	11.2 (4.8)
Semantic knowledge	38.7 (10.1)	39.6 (9.6)	40.6 (8.8)	39.7 (9.9)
Physical activity	1.7 (1.0)	1.6 (1.1)	1.5 (1.0)	1.6 (0.9)
Physical activity change	0.0 (0.0)	−0.2 (1.0)	−0.4 (1.0)	−0.4 (1.1)

M: mean, SD: standard deviation. The scoring ranges for each measure with a defined upper limit are as follows: education: 0−20, reasoning: 0−30, memory: 0−20, vocabulary: 0−36, physical activity: 0−4.

**Table 3:** SLS participant characteristics.

Measure	Year of testing			
	Baseline (*N* = 1658)	Year 7 (*N* = 940)	Year 14 (*N* = 447)	Year 21 (*N* = 181)
	M (SD)	M (SD)	M (SD)	M (SD)
Retention from previous testing (%)		56.7	47.6	40.5
Age	67.1 (8.2)	73.0 (7.3)	78.9 (6.4)	81.9 (4.9)
Education	14.6 (2.9)	14.7 (2.8)	14.8 (2.7)	14.8 (2.8)
Sex, female (*n* (%))	862 (52)	507 (54)	257 (57)	108 (60)
Reasoning	15.6 (5.8)	15.2 (5.6)	14.3 (5.5)	14.0 (5.3)
Fluency	38.5 (12.8)	37.5 (13.1)	36.7 (12.7)	38.8 (14.5)
Memory	12.5 (4.0)	12.0 (4.1)	11.5 (4.2)	11.7 (3.9)
Semantic knowledge	25.0 (6.7)	25.3 (6.6)	25.8 (6.2)	25.8 (5.9)
Physical activity	1.0 (1.1)	1.8 (1.1)	1.7 (1.0)	1.6 (0.9)
Physical activity Change	—	−0.1 (1.1)	−0.2 (1.1)	−0.0 (1.1)

M: mean, SD: standard deviation. The scoring ranges for each measure with a defined upper limit are as follows: education: 0–20, reasoning: 0–30, memory: 0–20, vocabulary: 0–36, physical activity: 0–4.

**Table 4:** VLS participant characteristics.

Measure	Year of testing						
	Baseline (*N* = 977)	Year 3 (*N* = 723)	Year 6 (*N* = 571)	Year 9 (*N* = 412)	Year 12 (*N* = 282)	Year 15 (*N* = 91)	Year 18 (*N* = 52)
	M (SD)	M (SD)	M (SD)	M (SD)	M (SD)	M (SD)	M (SD)
Retention from Previous testing (%)^a^	—	74	79	72	68	75	57
Age	68.6 (6.7)	71.3 (6.6)	73.7 (6.4)	76.5 (5.9)	79.3 (5.2)	82.2 (4.6)	85.1 (3.6)
Years of education at baseline	14.9 (3.3)	15.4 (3.2)	15.6 (3.1)	15.8 (3.1)	15.9 (3.1)	15.2 (3.1)	14.8 (2.8)
Sex, female (*n* (%))	614 (63)	450 (62)	348 (61)	252 (61)	177 (63)	60 (66)	35 (67)
Reasoning^b^	11.2 (4.5)	11.7 (4.2)	10.4 (4.7)	10.4 (4.6)	9.9 (4.6)	7.5 (4.7)	6.5 (4.2)
Fluency	17.7 (4.3)	17.9 (4.4)	17.8 (4.4)	17.2 (4.8)	16.3 (4.9)	14.7 (5.8)	13.8 (5.4)
Memory^c^	13.7 (5.9)	14.6 (6.0)	14.7 (6.2)	14.9 (6.4)	11.8 (5.5)	13.0 (6.3)	—
Semantic knowledge	43.7 (7.4)	44.7 (6.2)	44.3 (6.0)	44.2 (5.8)	43.5 (5.7)	42.7 (7.1)	42.6 (6.6)
Physical activity	15.4 (5.1)	15.4 (5.1)	15.0 (4.9)	14.8 (5.2)	13.4 (5.1)	14.0 (5.2)	12.3 (5.0)
Physical activity change	0.0 (0.0)	–0.1 (4.0)	–0.8 (4.2)	–1.3 (4.6)	–2.8 (4.8)	–2.7 (5.0)	–4.4 (5.2)

M: mean, SD: standard deviation. The scoring range for each measure with a defined upper limit are as follows: Reasoning 0–20, Fluency 0-maximum number of words produced in 6 minutes, Memory 0–30, Vocabulary 0–54. Physical Activity 0–36. The physical activity change scores are on a normal metric with means of approximately 0 and SD of approximately 0.8.

^a^The 1986 cohort was followed for up to 18 years, the 1993 cohort for up to 12.

^b^The reasoning measure was not given until year 6 for the 1986 cohort.

^c^The memory measure was not given in year 18.

**Table 5:** Mixed effects model summaries across four studies with baseline physical activity and activity change predicting four cognitive outcomes.

		OCTO-Twin			LBLS			SLS			VLS	
	*b*	SE	*P*	*b*	SE	*P*	*b*	SE	*P*	*b*	SE	*P*
Reasoning												
Intercept	10.66	0.56	<.001	21.53	0.56	<.001	37.88	1.02	<.001	9.53	0.59	<.001
Age	–0.37	0.12	0.002	–0.62	0.05	<.001	–0.35	0.01	<.001	–0.25	0.02	<.001
Sex	0.90	0.68	0.186	2.12	0.81	0.009	1.67	0.24	<.001	–0.01	0.31	0.969
Education	0.57	0.13	<.001	1.30	0.14	<.001	0.52	0.04	<.001	0.33	0.05	<.001
Activity	1.19	0.40	0.003	–0.50	0.40	0.209	–0.57	0.89	0.519	0.01	0.03	0.827
Age × activity	0.06	0.14	0.699	0.03	0.04	0.502	0.01	0.01	0.398	0.00	0.00	0.587
Education × activity	–0.09	0.13	0.479	–0.12	0.14	0.372	0.00	0.04	0.983	0.00	0.01	0.840
Time	–0.51	0.09	<.001	–0.46	0.09	<.001	0.15	0.10	0.141	–0.24	0.03	<.001
Age × time	–0.02	0.02	0.296	–0.02	0.01	0.001	–0.01	0.00	<.001	–0.01	0.00	<.001
Sex × time	0.13	0.10	0.206	0.09	0.11	0.445	0.02	0.03	0.318	0.00	0.03	0.968
Education × time	0.02	0.02	0.258	–0.06	0.02	0.007	–0.01	0.00	0.090	–0.01	0.00	0.065
Activity × time	0.06	0.07	0.354	0.04	0.06	0.506	0.01	0.01	0.183	0.00	0.00	0.332
Activity change	0.85	0.21	<.001	0.54	0.24	0.028	0.18	0.09	0.045	0.05	0.02	0.013

Fluency												
Intercept				31.19	0.62	<.001	58.66	2.55	<.001	11.73	0.56	<.001
Age				–0.25	0.05	<.001	–0.34	0.04	<.001	–0.10	0.03	<.001
Sex				4.25	0.90	<.001	3.54	0.59	<.001	0.46	0.36	0.200
Education				1.04	0.16	<.001	1.29	0.11	<.001	0.64	0.05	<.001
Activity				0.45	0.45	0.314	4.45	22.4	0.047	–0.06	0.03	0.098
Age × activity				0.07	0.05	0.159	–0.06	0.03	0.070	0.00	0.00	0.952
Education × activity				–0.29	0.15	0.059	–0.02	0.09	0.844	–0.01	0.01	0.159
Time				–0.27	0.11	0.013	0.78	0.23	0.001	–0.13	0.03	<.001
Age × Time				–0.03	0.01	0.001	–0.02	0.00	<.001	–0.01	0.00	0.002
Sex × Time				–0.22	0.15	0.132	0.13	0.05	0.007	0.07	0.04	0.070
Education × Time				0.00	0.03	0.983	–0.01	0.01	0.106	0.00	0.01	0.950
Activity × Time				0.03	0.08	0.680	0.05	0.02	0.048	0.01	0.00	0.045
Activity Change				0.05	0.35	0.894	0.41	0.20	0.043	0/07	0.03	0.003

Memory												
Intercept	8.59	0.31	<.001	10.78	0.20	<.001	23.50	0.74	<.001	17.36	0.42	<.001
Age	–0.22	0.06	<.001	–0.19	0.02	<.001	–0.18	0.01	<.001	–0.19	0.02	<.001
Sex	1.14	0.38	<.002	1.55	0.30	<.001	1.65	0.17	<.001	1.65	0.27	<.001
Education	0.39	0.07	<.001	0.29	0.05	<.001	0.32	0.03	<.001	0.32	0.04	<.001
Activity	0.48	0.22	0.003	0.15	0.15	0.312	–0.17	0.65	0.789	0.05	0.03	0.042
Age × Activity	–0.09	0.08	0.232	0.00	0.02	0.817	0.00	0.01	0.671	0.00	0.00	0.223
Education × Activity	0.02	0.07	0.837	–0.05	0.05	0.346	–0.03	0.03	0.215	0.00	0.01	0.607
Time	–0.32	0.07	0.001	–0.17	0.05	0.001	0.33	0.08	<.001	–0.27	0.03	<.001
Age × Time	–0.01	0.01	0.700	0.00	0.00	0.578	–0.01	0.00	<.001	–0.01	0.00	<.001
Sex × Time	0.08	0.08	0.319	–0.04	0.07	0.601	0.03	0.02	0.077	–0.02	0.03	0.469
Education × Time	0.01	0.02	0.972	0.01	0.01	0.379	0.00	0.00	0.449	–0.01	0.00	0.232
Activity × Time	–0.02	0.05	0.660	–0.02	0.04	0.531	0.02	0.01	0.078	0.00	0.00	0.202
Activity Change	0.44	0.15	0.004	–0.08	0.15	0.593	0.08	0.08	0.309	0.06	0.02	0.001

Semantic knowledge												
Intercept	30.21	0.85	<.001	38.36	0.56	<.001	23.41	1.29	<.001	43.86	0.71	<.001
Age	–0.60	0.17	<.001	–0.26	0.05	<.001	0.01	0.02	0.531	0.08	0.03	0.013
Sex	–3.88	1.04	<.001	1.47	0.81	0.069	0.56	0.30	0.069	0.02	0.47	0.971
Education	1.60	0.21	<.001	1.11	0.14	<.001	1.11	0.05	<.001	0.77	0.07	<.001
Activity	0.85	0.57	0.134	–0.54	0.40	0.174	1.61	1.14	–0.160	–0.02	0.04	0.716
Age × Activity	0.02	0.20	0.914	–0.02	0.04	0.672	–0.02	0.02	0.174	0.01	0.01	0.331
Education × Activity	–0.22	0.21	0.307	–0.31	0.14	0.023	–0.11	0.05	0.020	–0.01	0.01	0.588
Time	–0.93	0.12	<.001	–0.44	0.09	<.001	0.45	0.08	<.001	–0.14	0.03	<.001
Age × Time	–0.05	0.03	0.056	–0.04	0.01	<.001	–0.01	0.00	<.001	–0.01	0.00	<.001
Sex × Time	0.27	0.14	0.054	0.08	0.12	0.486	0.07	0.02	<.001	0.01	0.04	0.736
Education × Time	0.03	0.03	0.279	–0.03	0.02	0.134	0.00	0.00	0.792	–0.01	0.01	0.152
Activity × Time	–0.02	0.09	0.804	0.10	0.07	0.147	0.01	0.01	0.087	0.00	0.00	0.319
Activity Change	1.06	0.26	<.001	–0.04	0.25	0.881	0.11	0.08	0.148	0.02	0.02	0.258

Values represent model coefficients and their standard error. Across all studies, time was measured in years since baseline visit, and activity change was entered as a time-varying covariate. All other variables represent baseline measurements alone, in interaction with one another, or in interaction with time.
